# Polysaccharide Extracted from Longan (*Dimocarpus longan* Lour.) as Novel Adjuvant to Boost Humoral and Cellular Immune Responses

**DOI:** 10.3390/ijms27093980

**Published:** 2026-04-29

**Authors:** Da-Ping Xie, Zi-Hao Zhuang, Ming-Yu Jin, Ya-Hui Yu, Jing-Kun Yan

**Affiliations:** 1 Dongguan Key Laboratory of Typical Food Precision Design, China National Light Industry Key Laboratory of Healthy Food Development and Nutrition Regulation, School of Life and Health Technology, Dongguan University of Technology, Dongguan 523808, China; 19168860270@163.com (D.-P.X.); 17724298710@163.com (Z.-H.Z.); jinmingyu@dgut.edu.cn (M.-Y.J.); 2 College of Food Science, Henan University of Animal Husbandry and Economy, Zhengzhou 450046, China

**Keywords:** longan polysaccharide, adjuvant, toll-like receptor 4, humoral response, cellular response

## Abstract

Adjuvants, which enhance the effectiveness of antigens, are essential for vaccines against infectious or malignant diseases. Currently, the development of adjuvants encounters challenges as highly effective adjuvants tend to be highly toxic, whereas those with lower toxicity often lack efficacy. Polysaccharides have unique advantages as adjuvants due to their multiple immunomodulatory activities and favorable safety profiles. In this study, longan polysaccharide (LP) was characterized physicochemically and identified as an effective adjuvant. LP, consisting of 96.44% glucose, was mainly linked by the α-1,6-glycosidic bond. In vitro experiments revealed that LP could induce the secretion of pro-inflammatory cytokines (TNF-α, IL12, and IL1β) and expression of co-stimulatory molecules (CD80 and CD86) through toll-like receptor 4 (TLR4) activation. More importantly, LP could promote antigen cross-presentation when formulated with a model antigen—ovalbumin (OVA). In vivo experiments indicated that the LP+OVA formulation could boost both humoral and cellular immune responses in immunized C57BL/6J mice. The histopathological evaluation of the major organs showed that LP+OVA was non-toxic. Therefore, our findings suggested that LP is an effective and safe adjuvant for vaccine development.

## 1. Introduction

Vaccines hold great promise in combating infections and malignant diseases by training the immune system to recognize and respond effectively to specific pathogens or cancer cells, thus reducing the morbidity and mortality associated with these conditions [1,2]. A central objective of contemporary vaccine design is to elevate antigen immunogenicity, a function for which adjuvants are specifically developed [3]. The first adjuvant was Coley’s toxins, a mixture of heat-killed bacteria (*Streptococcus pyogenes* and *Serratia marcescens*), which can be tracked back to the early 1890s [4]. However, to date, only a small number of adjuvants (e.g., aluminum hydroxide, MF59, CpG 1018, AS01, AS03 and AS04) have been approved for clinical use due to challenges such as low efficiency, severe side effects, and a limited understanding of their mechanism of action [5,6]. These commercial adjuvants could induce robust immune response by activating antigen-processing cells (APCs), boosting the immune response of antigens in a relatively safe way. However, they are still unable to fully meet the clinical requirements. For instance, aluminum adjuvants tend to induce a Th2 instead of Th1 immune response and the mechanism is still unclear [7]. More importantly, experimental research has clearly shown that aluminum adjuvants have a risk of autoimmunity, long-term brain inflammation, and associated neurological complications, and may thus have profound and widespread adverse health consequences [8]. Adjuvant systems that integrate different kinds of adjuvants also have disadvantages such as a complex preparation process and instability [9]. Therefore, developing highly effective, low-toxicity adjuvants with a clearly defined mechanism of action holds significant clinical importance.

Polysaccharides have unique advantages when serving as adjuvants, owing to their substantial abundance, low cost, high compatibility, chemical versatility, and most importantly, immunomodulatory activities [10,11]. Immune cells have developed a variety of pattern recognition receptors (PRRs), which allow them to identify pathogen-associated molecular patterns (PAMPs) [12,13]. The carbohydrate signals of bacteria and fungi represent the most common PAMPs [14]. Once these signals are detected, PRRs trigger signaling cascades to activate immune cells. This activation stimulates the production of pro-inflammatory cytokines, chemokines, and other mediators, which jointly coordinate a rapid immune defense against infection [15]. For instance, a lipopolysaccharide (LPS) derived from Gram-negative bacteria can be recognized by toll-like receptor 4 (TLR4) [16], and zymosan obtained from yeast can be recognized by dectin-1 and toll-like receptor 2 (TLR2) [17]. However, the application of these microbial-derived polysaccharides is restricted due to high toxicity and batch-to-batch variability [18,19]. An alternative is a polysaccharide from a non-microbial origin, which can also mimic such effects and be produced on a large scale. For instance, inulin from *Chicory*, *Jerusalem artichoke*, and *Dahlia* can enhance both humoral and cellular immune responses to the trivalent influenza vaccine in intramuscularly immunized mice without causing significant inflammation [20,21]. Notably, polysaccharides derived from Chinese medicinal herbs exhibit an unexpected adjuvant effect. For example, *Astragalus* polysaccharides have been shown to effectively elevate the levels of antibodies specific to avian infectious bronchitis virus [22]. *Lycium barbarum* polysaccharide, when formulated with an antigen, can induce expressions of TNF-α and IFN-γ and promote antigen-specific humoral immune response [23]. Therefore, these findings inspired us to contemplate polysaccharides derived from Chinese medicinal herbs as potential sources for the development of adjuvants.

Longan (*Dimocarpus longan* Lour.), a tropical fruit belonging to the *Sapindaceae* family, is primarily cultivated in Asian regions (e.g., China, Thailand, and Vietnam). Dried longan pulp has been used as a traditional Chinese medicine due to its immunomodulatory effect and is included in the “Food-Medicine Homology” list [24]. Recent studies have revealed that longan polysaccharide (LP) is the principal bioactive component of the fruit, possessing the ability to activate macrophages [25] and induce the differentiation of Th1 and Treg cells [26]. This finding inspired us to consider whether LP could be an ideal candidate as an adjuvant. However, the adjuvant activities of LP have been scarcely investigated, and the mechanism of action remains unclear.

In this study, therefore, LP was extracted through a magnetic-induced electric field (MIEF)-assisted three-phase partitioning method (TPP) and the physicochemical properties of LP were characterized by size-exclusion chromatography (SEC), Fourier transform infrared (FTIR) spectroscopy and nuclear magnetic resonance (NMR) analysis. Bone marrow-derived dendritic cells (BMDCs) were used to assess the effect of LP on DC maturation. After confirming that LP could promote the maturation of DCs through TLR4 activation, we combined LP with a model antigen, ovalbumin (OVA), to formulate a vaccine (LP+OVA), and the cross-presentation efficiency of this vaccine was detected. Encouraged by in vitro results, LP+OVA was utilized to immunize C57BL/6J mice. The humoral responses were assessed by determining the OVA-specific antibody via enzyme-linked immunosorbent assay (ELISA) and the cellular responses were evaluated by quantifying the proportion of IFN-γ^+^ cells in CD4^+^ and CD8^+^ T cells after restimulation with corresponding OVA-specific peptide. Histopathological analysis of the major organs was performed to evaluate the toxicity of LP as an adjuvant.

## 2. Results

### 2.1. Structural Characterization of LP

**Figure 1:**
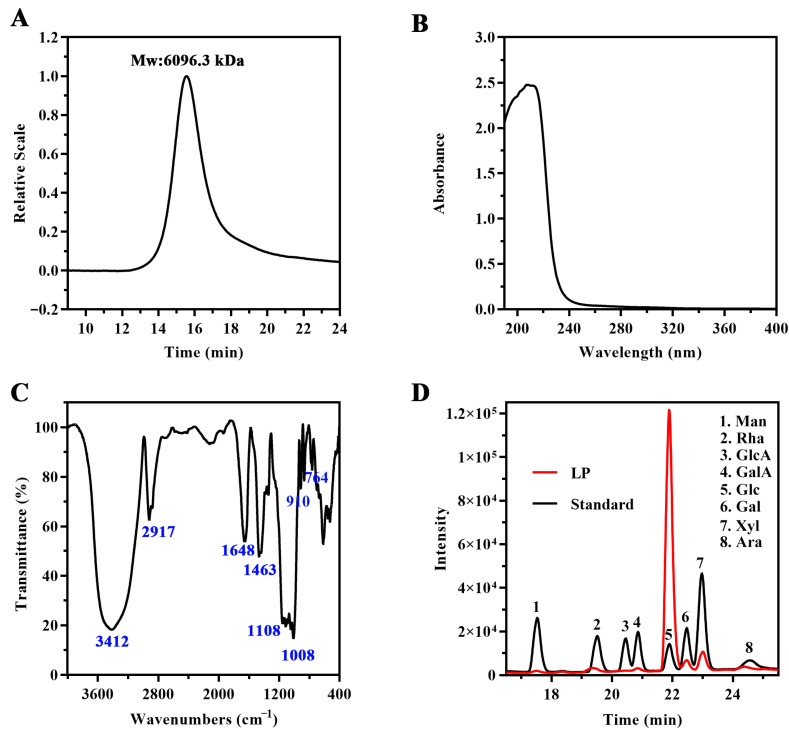
Characterization of LP. (**A**) Size exclusion chromatograms of LP. (**B**) UV spectrum of LP. (**C**) IR spectrum of LP. (**D**) HPLC analysis of monosaccharides in standard sample and LP.

Longan polysaccharide (LP) was extracted and isolated from the pulp of *Dimocarpus longan* Lour using the MIEF-TPP method, with an obtained yield of 3.55%. The SEC profile of LP exhibited a single peak, suggesting a relatively uniform molecular weight (Mw: 6096.3 kDa; Mn: 5759.4 kDa; Mw/Mn = 1.06) (Figure 1A). The UV spectrum of LP (Figure 1B) exhibited a downward trend with increasing wavelength, consistent with the typical characteristics of polysaccharides. The FTIR spectrum of LP exhibited band characteristics that are typical of polysaccharides, including the stretching vibration of a hydroxyl group (-OH) at 3412 cm^−1^, the stretching vibration of C-H at 2917 cm^−1^, the stretching vibrations of a carbonyl group at 1648, 1463 and 1339 cm^−1^, and the stretching vibration of C-O (glycosidic linkage) at 1108 and 1008 cm^−1^. The absorption peaks at 910 and 764 cm^−1^ belong to the antisymmetric ring vibration and the symmetrical ring vibration of a D-glucopyranose ring, respectively (Figure 1C). Monosaccharide composition analysis revealed that LP was composed of mannose (Man), rhamnose (Rha), galacturonic acid (GalA), glucose (Glc), galactose (Gal), xylose (Xyl), and arabinose (Ara), with a relative molar ratio of 0.37:0.88:0.30:96.17:0.93:1.26:0.11, indicating that Glc was the predominant monosaccharide in LP (Figure 1D).

**Figure 2:**
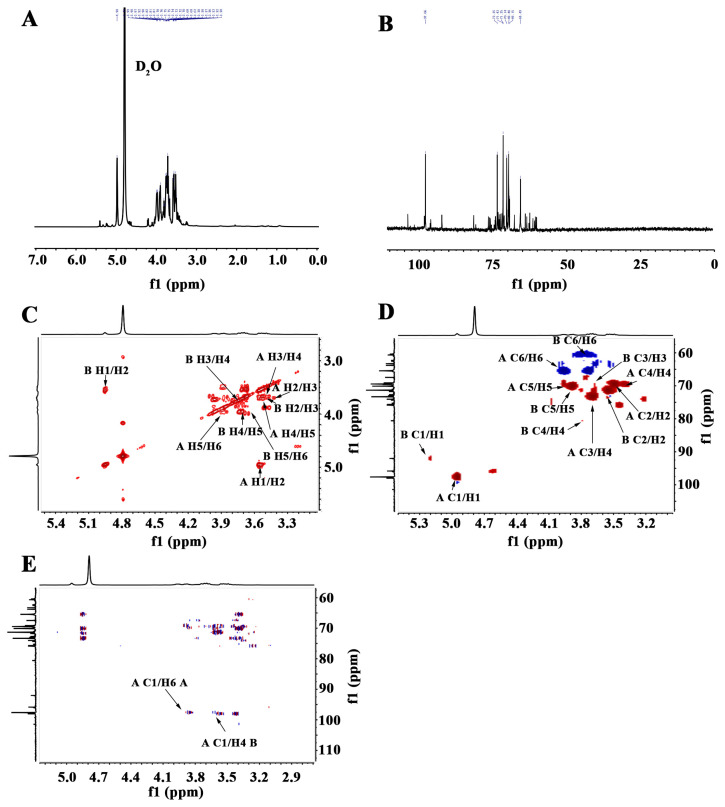
Structural analysis of LP. (**A**) ^1^H NMR spectrum of LP. (**B**) ^13^C NMR spectrum of LP. (**C**) ^1^H-^1^H COSY spectrum of LP. (**D**) HSQC spectrum of LP. (**E**) HMBC spectrum of LP.

NMR analysis was conducted to characterize the structure of LP. The ^1^H NMR spectrum revealed two main anomeric proton signals at chemical shifts of 5.22 and 4.95 ppm (Figure 2A). The ^13^C NMR spectrum displayed signals corresponding to two anomeric carbons at chemical shifts of 98.00 and 97.81 ppm. Signals within the chemical shift range from 69.12 to 75.87 ppm were attributed to downfield shifts resulting from substitutions at C-2, C-3, or C-4 by glycosidic residues (Figure 2B). The main sugar residues were assigned as residue A (δ 4.95 ppm) and residue B (δ 5.22 ppm) (Table S1, Supplementary Material). The ^1^H-^1^H COSY spectrum indicated connections from the H-1 signal at δ 4.95 ppm to the H-2 signal at δ 3.56 ppm and the H-3 signal at δ 3.69 ppm; from the H-3 signal to the H-4 signal at δ 3.49 ppm and the H-5 signals at δ 3.89 ppm; and from the H-5 signal to the H-6 signal at δ 3.97/3.71 ppm (Figure 2C). Further analysis of the HSQC spectrum allowed us to identify the corresponding anomeric proton signals. In the HSQC spectrum, the C-H correlation signals of C-6 of aldohexoses were observed: δ 60.66, 3.72/3.79 ppm; 63.65, 3.42 ppm; 65.45, 3.87 ppm; and 65.40/3.64, 3.7/3.61 ppm. The downfield shift of C-6 of aldohexoses at δ 69.44 ppm indicated that some glycosidic residues were substituted at C-6 (Figure 2D). In the HMBC spectrum, the C-1 signal peak of residue A in LP exhibited coupling with the H-6 signal peak, indicating that C-1 in residue A is linked to its C-6 position. The signal peaks of C-1 (δ 98.00 ppm) and H-6 (δ 3.86 ppm) in residue A suggest that it is connected to itself via α-1,6-glycosidic bonds, thereby forming a structure of residue A (→6)-α-Glcp-(1→). Additionally, the C-1 signal peak in residue A showed coupling with the H-4 signal peak in residue B, as confirmed by the C-1 (δ 97.81 ppm) and H-6 (δ 3.59 ppm) signal peaks, indicating that C-1 of residue A is attached to the O-4 position of residue B (→4)-α-Glcp-(1→) (Figure 2E).

### 2.2. LP Stimulated DCs to Express and Secrete Pro-Inflammatory Cytokines

**Figure 3:**
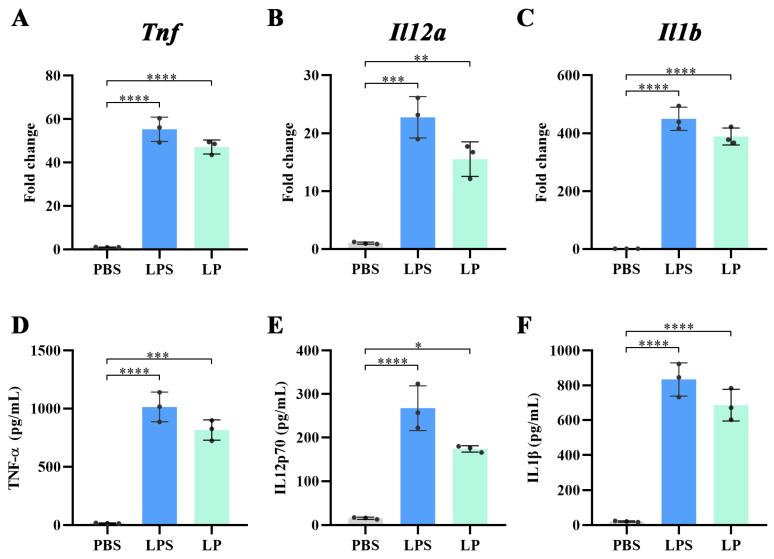
LP promotes the expression of pro-inflammatory cytokines by DCs. (**A**–**C**) Gene expression of *Tnf* (**A**), *Il12a* (**B**) and *Il1b* (**C**) in BMDCs treated with LP at a concentration of 100 μg/mL for 24 h. LPS was used as a control, with a concentration of 100 ng/mL. (**D**–**F**) Secretion of TNF-α (**D**), IL12p70 (**E**) and IL1β (**F**) by BMDCs treated with LP at a concentration of 100 μg/mL for 24 h. LPS was used as a control, with a concentration of 100 ng/mL. *n* = 3; values represent means ± standard deviation. Statistical significance was calculated by one-way ANOVA with Tukey’s post hoc test. ns: no significance; * *p* < 0.05, ** *p* < 0.01, *** *p* < 0.001, and **** *p* < 0.0001.

The viability of BMDCs treated with LP was assessed. As demonstrated in Figure S1 (Supplementary Material), exposure of BMDCs to LP at concentrations ranging from 0 to 1000 µg/mL did not elicit any toxic effects. To investigate the potential of LP to promote DC maturation, BMDCs were treated with LP for 24 h. LPS was used as a positive control. The qPCR results demonstrated that LP significantly enhanced the mRNA expression of pro-inflammatory factors. Specifically, there was a 46.2-fold increase in *Tnf* (*p* < 0.0001), a 14.5-fold increase in *Il12a* (*p* < 0.01), and a remarkable 387.9-fold increase in *Il1b* (*p* < 0.0001) when compared to BMDCs treated with PBS (Figure 3A–C). The secretion of the corresponding cytokines was determined by ELISA. As shown in Figure 3D–F, LP significantly elevated the levels of pro-inflammatory cytokines, leading to a 52.7-fold increase in TNF-α (*p* < 0.001), a 10.2-fold increase in IL-12p70 (*p* < 0.05), and a 32.3-fold increase in IL1β (*p* < 0.0001), respectively. The ELISA data was consistent with the qPCR results, suggesting that LP could promote the expression of pro-inflammatory cytokines.

### 2.3. LP Induced BMDC Maturation by Activating TLR4

**Figure 4:**
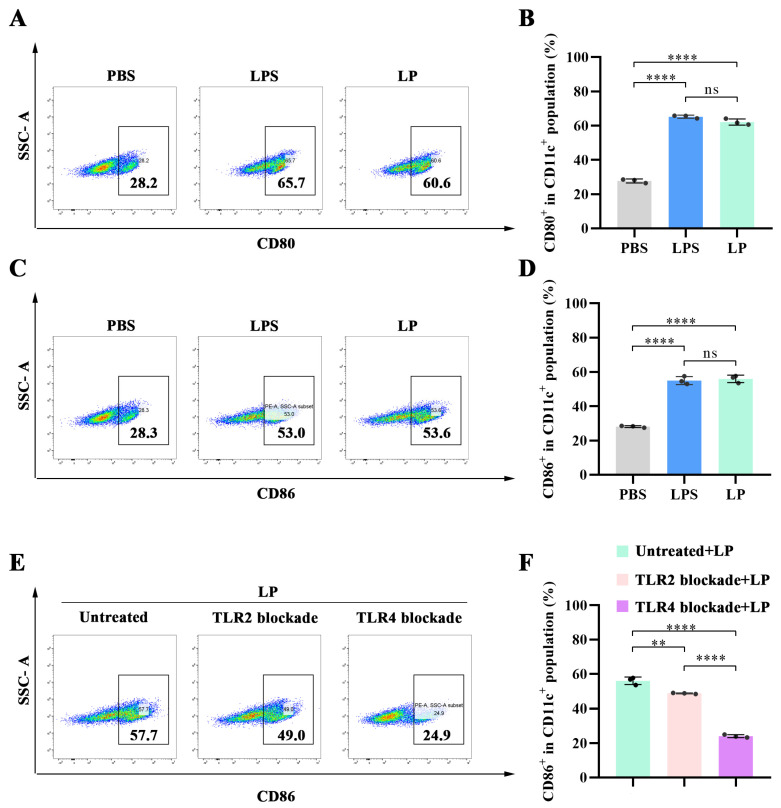
LP promotes DC maturation via TLR4. (**A**) Representative flow cytometry plots of CD80 expression of BMDCs treated with LPS at a concentration of 100 ng/mL or LP at a concentration of 100 μg/mL for 24 h. (**B**) Proportion of CD80^+^ cells in CD11c^+^ cells treated with LPS at a concentration of 100 ng/mL or LP at a concentration of 100 μg/mL for 24 h. (**C**) Representative flow cytometry plots of CD86 expression of BMDCs treated with LPS at a concentration of 100 ng/mL or LP at a concentration of 100 μg/mL for 24 h. (**D**) Proportion of CD86^+^ cells in CD11c+ cells treated with LPS at a concentration of 100 ng/mL or LP at a concentration of 100 μg/mL for 24 h. (**E**) Representative flow cytometry plots of CD86 expression of BMDCs pretreated with TLR2- or TLR4-blocking antibody for 1 h, followed by LP treatment at a concentration of 100 μg/mL for 24 h. (**F**) Proportion of CD86^+^ cells in CD11c^+^ cells pretreated with TLR2- or TLR4-blocking antibodies for 1 h, followed by LP treatment at a concentration of 100 μg/mL for 24 h. *n* = 3; values represent means ± standard deviation. Statistical significance was calculated by one-way ANOVA with Tukey’s post hoc test. ns: no significance; ** *p* < 0.01 and **** *p* < 0.0001.

To further assess the potential of LP as an adjuvant, the expression of co-stimulatory factors (e.g., CD80 and CD86) was determined using flow cytometry. LPS was used as positive control. As shown in Figure 4A,B, LP significantly increased the proportion of CD80^+^ cells within the CD11c^+^ population, rising from 27.70% to 62.13% (*p* < 0.0001). In parallel, the proportion of CD86^+^ in CD11c^+^ cells was also elevated, increasing from 28.10% to 56.03% (*p* < 0.0001) (Figure 4C,D). The qPCR results also verified this point (Figure S2, Supplementary Material). These results, in conjunction with the expression of pro-inflammatory cytokines, suggested that LP could promote DC maturation.

Subsequently, we investigated which signaling pathway is predominantly involved in LP-induced DC maturation. Numerous studies have demonstrated that the carbohydrate signals of polysaccharides are typically recognized by TLRs or C-type lectin receptors (CLRs), especially TLR [11]. Therefore, we pretreated BMDCs with TLR2- and TLR4-blocking antibodies for 1 h before LP stimulation. As shown in Figure 4E,F, blocking TLR2 slightly reduced the expression of CD86 (*p* < 0.01), whereas blocking TLR4 significantly reduced the expression of CD86 (*p* < 0.0001). This suggested that LP-induced DC maturation is mainly attributed to TLR4. Collectively, these data indicated that LP induces DC maturation mainly through the activation of TLR4.

### 2.4. LP Enhanced the Cross-Presentation of Antigens In Vitro

**Figure 5:**
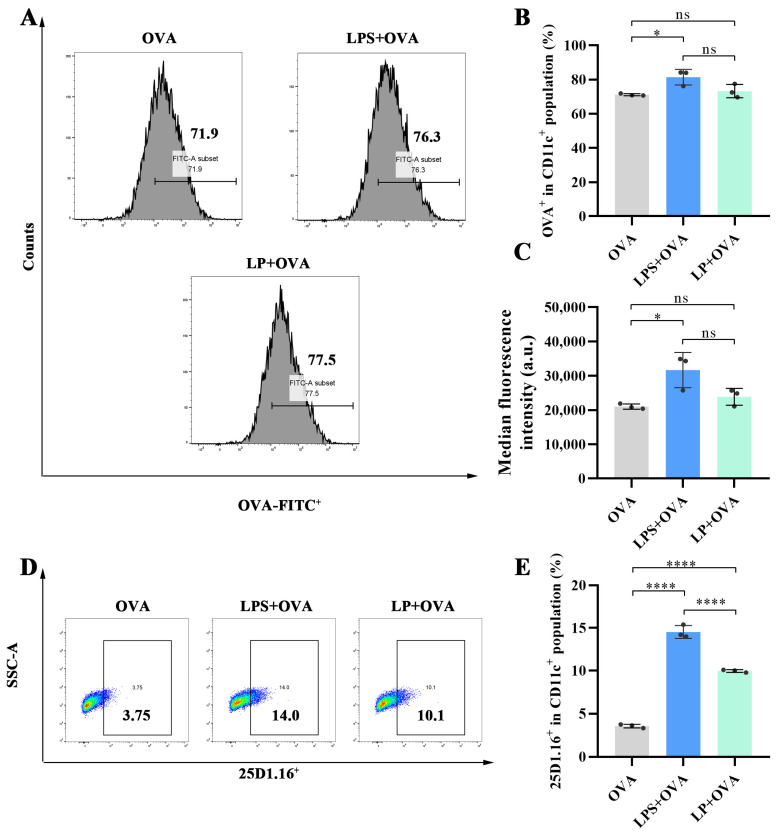
LP enhances cross-presentation of antigens. (**A**) Representative flow cytometry plots of cellular uptake of OVA by BMDCs treated with LPS or LP. (**B**) Proportion of OVA-FITC^+^ BMDCs after treatment with OVA, LPS+OVA or LP+OVA. (**C**) Median fluorescence intensity of OVA-FITC^+^ BMDCs. (**D**) Representative flow cytometry plots of cross-presentation of antigens by BMDCs treated with OVA, LPS+OVA or LP+OVA after 16 h incubation. (**E**) Proportion of 25D1.16^+^ BMDCs after treatment with OVA, LPS+OVA or LP+OVA after 16 h incubation. *n* = 3; values represent means ± standard deviation. Statistical significance was calculated by one-way ANOVA with Tukey’s post hoc test. ns: no significance; * *p* < 0.05 and **** *p* < 0.0001.

As illustrated above, LP could promote the maturation of DCs through the activation of TLR4. The maturation of DCs plays a crucial role in connecting innate immunity and adaptive immunity. Mature DCs can process and present antigens to T cells, thereby initiating an adaptive immune response. Therefore, we investigated the effects of LP on antigen phagocytosis and cross-presentation. As shown in Figure 5A–C, compared to OVA, the LPS+OVA could increase the proportion of FITC-OVA^+^ cells in CD11c^+^ cells (*p* < 0.05), suggesting that LPS could enhance antigen phagocytosis to a certain extent. In contrast, LP had little effect on OVA uptake, and this effect was not statistically significant (*p* > 0.05). Since the activation of TLR4 by LPS has been demonstrated to cross-present antigens, we investigated whether LP could achieve such a function. The specific monoclonal antibody 25D1.16, which specifically binds to the mouse MHC class I molecule H-2Kb when presenting the OVA peptide SIINFEKL (OVA 257-264), was employed. As shown in Figure 5D–E, the proportion of 25D1.16^+^ cells in BMDCs treated with LP+OVA was significantly higher than that in those treated with OVA (14.53% vs. 9.96%, *p* < 0.0001), suggesting that LP indeed promoted the cross-presentation of antigens.

### 2.5. LP Boosted Both Humoral and Cellular Immune Responses

**Figure 6:**
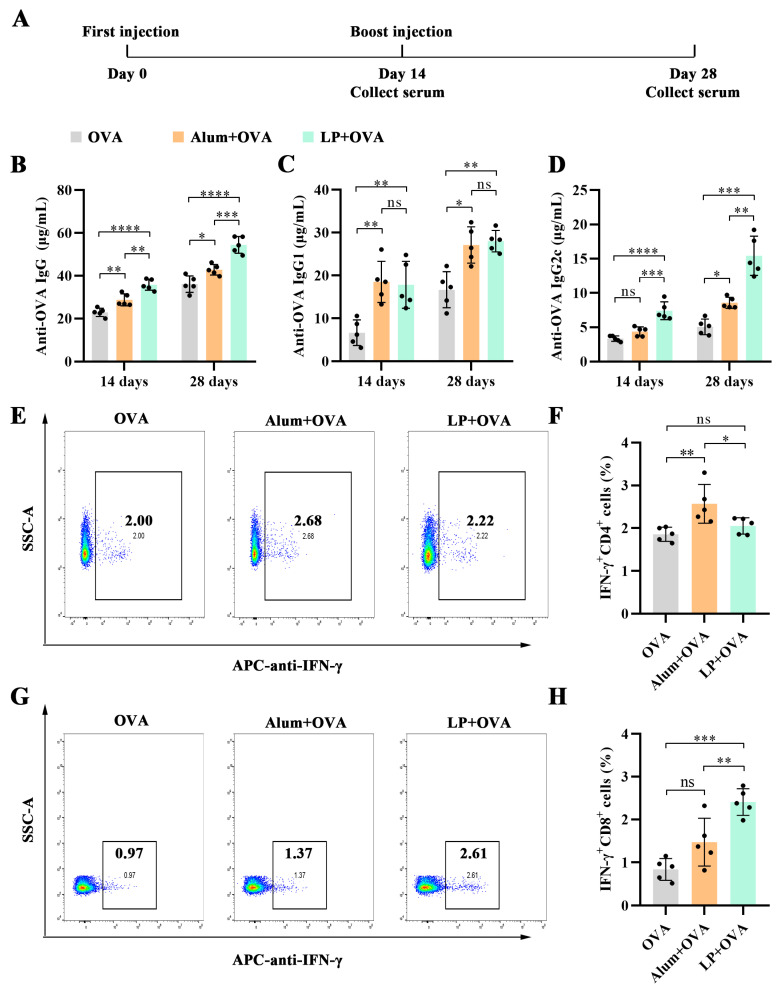
LP could boost both humoral and cellular immune responses. (**A**) Diagram of experimental design. On Day-0 and Day-14, mice received the first and booster immunization, respectively. (**B**) The levels of anti-OVA IgG antibodies in the serum of mice treated with OVA, Alum+OVA or LP+OVA on Day-14 and Day-28. (**C**) The levels of anti-OVA IgG1 antibodies in the serum of mice treated with OVA, Alum+OVA or LP+OVA on Day-14 and Day-28. (**D**) The levels of anti-OVA IgG2c antibodies in the serum of mice treated with OVA, Alum+OVA or LP+OVA on Day-14 and Day-28. (**E**,**F**) Percentage of CD4^+^ T cells expressing IFN-γ in the spleen after stimulation with OVA323-339. (**G**,**H**) Percentage of CD8^+^ T cells expressing IFN-γ in the spleen after stimulation with OVA257-264. n = 5; values represent means ± standard deviation. Statistical significance was calculated by one-way ANOVA with Tukey’s post hoc test. ns: no significance; * *p* < 0.05, ** *p* < 0.01, *** *p* < 0.001, and **** *p* < 0.0001.

On Day-0 and Day-14, mice were subcutaneously immunized via hind footpad injection using an insulin syringe. Aluminum hydroxide formulated with OVA (Alum+OVA) was used as a positive control. Serum samples were collected on Day-14 and Day-28 to evaluate humoral immunity by measuring the levels of OVA-specific IgG, IgG1, and IgG2c (Figure 6A). As shown in Figure 6B–D, both Alum+OVA and LP+OVA elicited significantly higher antibody responses compared to OVA on Day-14. Booster immunization further enhanced the production of antibodies on Day-28. Notably, LP+OVA induced higher IgG antibodies than Alum+OVA (*p* < 0.01 on Day-14; *p* < 0.001 on Day-28), suggesting that LP was more effective than aluminum hydroxide in the induction of humoral response. No difference between LP+OVA and Alum+OVA in IgG1 production was observed on either Day-14 or Day-28 (Figure 6C). Alum+OVA had little effect on IgG2c production (Figure 6D). In contrast, LP+OVA induced higher IgG2c antibody production than Alum+OVA, indicating that LP could induce a Th1-biased immune response. These findings suggested that LP holds promise as a novel adjuvant candidate for vaccines due to its capacity to boost humoral immune response.

To investigate the T cell response induced by LP+OVA, splenocytes were harvested from vaccinated mice on Day-56 and subsequently stimulated with an OVA-specific, MHC-I-restricted peptide (OVA 257-264: SIINFEKL) or an OVA-specific, MHC-II-restricted peptide (OVA 323-339: ISQAVHAAHAEINEAGR) for 24 h. The proportions of interferon-γ (IFN-γ)-positive CD4^+^ and CD8^+^ T cells were assessed by flow cytometry, respectively. As shown in Figure 6E,F, CD4^+^ T cells from mice treated with Alum+OVA tended to produce more IFN-γ than those from mice treated with OVA (*p* < 0.01) or LP+OVA (*p* < 0.05). In contrast, LP+OVA showed no ability to induce more IFN-γ^+^ CD4^+^ T cells compared to OVA. It is worth noting that LP+OVA could significantly enhance the production of IFN-γ in CD8^+^ T cells, compared with OVA (*p* < 0.001), with the proportion increasing from 0.84% to 2.41%. However, Alum+OVA failed to induce IFN-γ production in CD8^+^ T cells (Figure 6G,H). These data revealed that LP+OVA could induce an efficient CD8^+^ T cell-mediated immune response.

### 2.6. In Vivo Safety Evaluation

**Figure 7:**
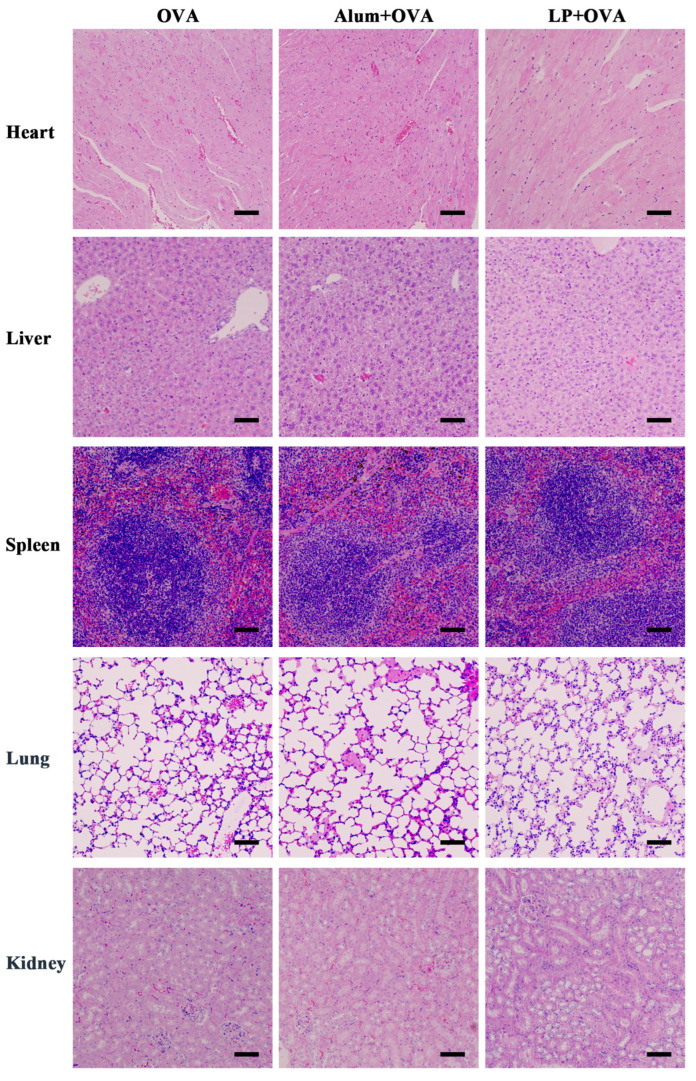
Histological assessment of the heart, liver, spleen, lung, and kidney of mice treated with OVA, Alum+OVA or LP+OVA. Mice were subcutaneously injected with OVA, Alum+OVA or LP+OVA on Day-0 and Day-14. On Day-56, the mice were sacrificed, and main organs were collected for paraffin sectioning and H&E staining. Scale bar: 200 μm.

A histopathological evaluation of the major organs was conducted using hematoxylin and eosin (H&E) staining to assess the potential toxicity of the LP+OVA treatment. Organs, including the heart, liver, spleen, lungs, and kidneys, were collected from the immunized mice on Day-56. As shown in Figure 7, no pathological abnormalities were observed across all treatment groups. In cardiac tissues, both the myocardium and epicardium displayed normal morphology, devoid of any indications of damage or inflammation. Splenic architecture remained preserved, with the ratio of red pulp to white pulp falling within normal physiological ranges. Renal examination revealed typical glomerular and tubular structures, without any alterations in the number or volume of glomeruli or renal tubular epithelium. Furthermore, no evidence of toxicity or adverse histological changes was observed in the lung and liver tissues of mice immunized with LP+OVA. Collectively, these findings indicated that immunization with LP+OVA did not lead to detectable histopathological damage to the major organs.

## 3. Discussion

**Figure 8:**
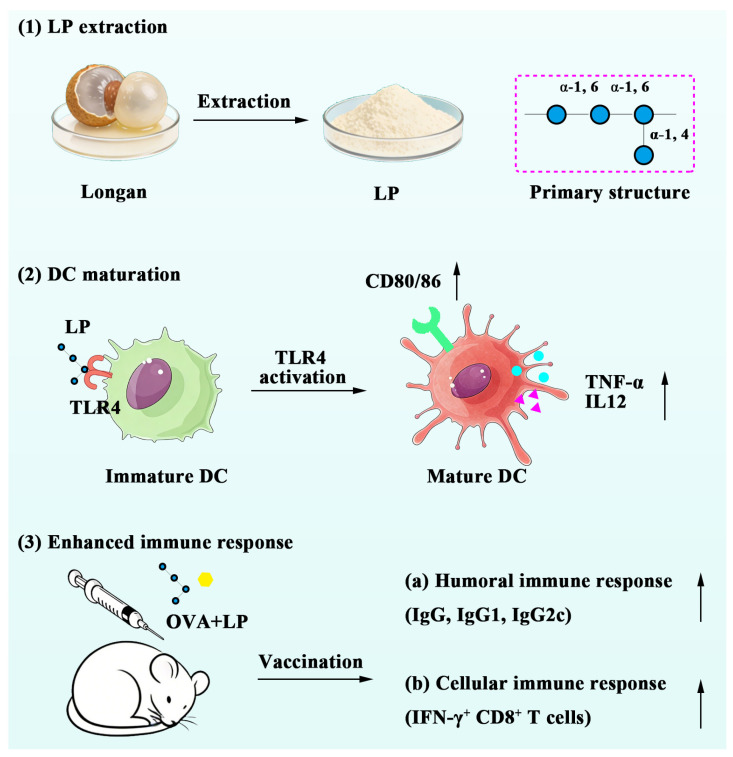
Schematic illustration of LP as a novel adjuvant to boost immune response of vaccines.

The dried pulp of longan fruit (Longan aril) has been highly regarded within the paradigm of the “Food-Medicine Homology” concept for decades, owing to its health-protecting effects [27]. In this work, as illustrated in Figure 8, we extracted a polysaccharide from longan and demonstrated that longan polysaccharide can significantly promote the expression of CD80 and CD86, as well as the secretion of pro-inflammatory cytokines (TNF-α and IL12), in BMDCs via TLR4 activation. These mediators serve as co-stimulation signals and differential signals for T cell activation, respectively. Crucially, LP exhibited no toxicity during both in vitro and in vivo experiments, which underscores its biocompatibility. When formulated with OVA, the LP+OVA could generate both humoral and cellular immune responses, thereby confirming the adjuvant activities of LP. Current studies have revealed that LP serves as a stimulator for macrophages to produce NO [25] and a mediator for the differentiation of T cells by DCs [26], while direct adjuvant activities of formulation (LP+OVA) have not been investigated.

Ample evidence has revealed that polysaccharides, such as lentinan from *Shiitake*, Astragalus polysaccharide and β-glucan from yeast, are potential adjuvants [28,29,30]. The common feature of these polysaccharides is that the main linkage is β-1,3 with or without β-1,6 branches, which rarely exist in mammals. Various investigations have demonstrated that some polysaccharides could activate two or more receptors [31]. In this work, LP was predominantly linked by the α-1,6 glycosidic bond, which is common in mammals. Under such conditions, LP could be hydrolyzed more easily than other polysaccharides. In addition, LP specifically activates TLR4. Therefore, LP is safer than other polysaccharide adjuvants. More importantly, the safety of LP could partially be attributed to the source, which has been included in “Food-Medicine Homology” and verified for years.

Many physicochemical factors intricately govern the biological activity of carbohydrates and their recognition by PRRs [32,33]. Key factors include monosaccharide composition, molecular weight, primary linkage patterns, and higher-order conformational structures. First, the monosaccharide composition serves as a fundamental identity code. Certain sugars, such as rhamnose, galacturonic acid, and arabinose, exist in LP but are rare in mammalian glycans. These “non-self” sugar residues may function as critical epitopes for immune recognition [34]. Second, molecular mass and structural repetitiveness are pivotal for effective receptor engagement and signaling initiation. As a high-molecular-weight polysaccharide, LP possesses an extended chain with highly repetitive structural units, allowing a single LP molecule to simultaneously bind to multiple TLR4 receptors on the cell membrane. Such multivalent binding is crucial for inducing receptor clustering, which overcomes the low affinity typically observed in single protein–carbohydrate interactions [35,36]. Finally, the conformation, largely influenced by the branching pattern, determines the spatial fitness for receptor binding [37]. A relatively linear or less-branched structure, as suggested for LP, facilitates the polymer chain in adopting an orderly and stable spatial conformation, such as an extended helix or a folded ribbon. Such a well-defined three-dimensional shape is more likely to present a continuous and complementary surface for interaction with the specific pocket or interface on TLR4.

TLR activation has been demonstrated to enhance antigen cross-presentation in vitro and in vivo [38,39,40]. Notably, such a process is delicately controlled by the NADPH oxidase 2 (NOX2)/reactive oxygen species (ROS) axis [41]. For instance, upon stimulation with LPS, DCs could recruit NOX2 to antigen-containing phagosomes, which led to the sustained production of ROS [42]. This process results in the consumption of protons within the phagosome, alkalinizing the acidic environment to prevent excessive degradation of antigens, thus promoting antigen presentation. In this study, LP exhibiting TLR4 agonistic activity was also capable of cross-presenting the epitopes of OVA, similarly to LPS. However, whether NOX2 is involved in LP-mediated cross-presentation was not investigated in this work. Further studies will focus on the influence of LP on NOX2 recruitment as well as antigen processing.

## 4. Materials and Methods

### 4.1. Materials and Reagents

*Dimocarpus longan* Lour. were purchased from Maoming, Guangdong Province. Fetal bovine serum and RPMI 1640 medium were obtained from Gibco (Waltham, MA, USA). Ovalbumin (OVA) and penicillin–streptomycin (100×) were sourced from Sigma-Aldrich (St. Louis, MO, USA). Phycoerythrin (PE)-conjugated anti-CD80 antibody, PE-conjugated anti-CD86 antibody, PE-Cy7-conjugated anti-CD11c antibody, Allophycocyanin (APC) anti-mouse H-2K bound to SIINFEKL antibody, and APC-conjugated anti-IFN-γ antibody were purchased from BioLegend (San Diego, CA, USA). OVA, OVA 257-264, and OVA 323-339 were purchased from Sangon Biotech (Shanghai, China). Aluminum hydroxide adjuvant was purchased from MedChemExpress (Monmouth Junction, NJ, USA). All other chemicals and reagents were acquired from Aladdin (Shanghai, China).

### 4.2. Cells and Animals

BMDCs were isolated from the bone marrow of 6–8-week-old female C57BL/6J mice and cultured in RPMI 1640 medium supplemented with 10% inactivated fetal bovine serum and 1% penicillin–streptomycin. GM-CSF (20 ng/mL) and IL4 (10 ng/mL) were added every 2 days to induce BMDC differentiation.

All mice were housed in a Specific Pathogen-Free (SPF) facility and maintained under controlled conditions of light (12 h light–dark cycles), humidity, and temperature. They were fed with a standard chow diet and water. The experimental procedures involving animals were reviewed and approved by the Dongguan University of Technology Animal Care and Use Committee (Approval No. DGUT202503030), complying with the Guidelines for the Care and Use of Laboratory Animals published by the National Institutes of Health, USA.

### 4.3. Extraction and Isolation of LP

The LP was prepared through the MIEF-TPP method [43]. Briefly, 200 g of fresh longan was mixed with 1.5 L of distilled water. The pH value was adjusted to 5.5 by 1.0 mol/L HCl, and the mixture was subsequently processed using a MIEF system (INDUC Scientific, MIH-P10, Wuxi, China) at an excitation voltage of 400 V for 90 min. Then the reaction system was cooled, and the pH value was adjusted to 7.0 by 3.0 mol/L NaOH solution. The supernatant was collected via centrifugation (8000× *g*, 10 min) and concentrated into one-tenth volume (150 mL) using a vacuum rotary evaporator. After that, ammonium sulfate was added to the concentrate to a final concentration of 20% (*w*/*v*), followed by mixing with n-butyl alcohol at a volume ratio of 1:1.5 (*v*/*v*). After stirring and centrifugation, the lower aqueous phase containing crude polysaccharide was collected. The aqueous phase containing crude polysaccharide was then dialyzed against dd-H_2_O using a molecular weight cutoff of 7000 Da for 48 h and lyophilized to obtain LP solid.

### 4.4. Characterization of LP

*Molecular weight determination*: The molecular weight of LP was determined using size-exclusion chromatography coupled with multi-angle laser light scattering (SEC-MALLS). The system consisted of an OmniSEC chromatograph (OmniSEC, Agilent, Santa Clara, CA, USA) which was connected to an Eclipse DualTec MALLS detector (Eclipse DualTec, Wyatt, Santa Barbara, CA, USA) equipped with a refractive index (RI) detector. Separation was performed on a Shodex OHpak LB-806 M column (8 mm × 30 cm) with 0.1 M NaCl as the mobile phase at a flow rate of 0.5 mL/min. The sample injection volume was 100 μL. The refractive index increment (dn/dc) for LP was measured at 657 nm and determined to be 0.145 mL/g.

*Ultraviolet–visible spectroscopy*: LP was dissolved in distilled water at a concentration of 0.5 mg/mL. Distilled water was used as the blank control. The ultraviolet absorption spectrum of LP was recorded on a UV–visible spectrophotometer over the wavelength range of 190-400 nm using a quartz cuvette with a 1 cm path length.

*FTIR*: LP (2.0 mg) was thoroughly ground with KBr (200 mg) and pressed into a transparent pellet. The Fourier transform infrared (FTIR) spectrum of LP was recorded on an FTIR spectrometer (Nicolet iS50, Thermo Fisher Scientific, Waltham, MA, USA) within the range of 4000–400 cm^−1^ at a resolution of 4 cm^−1^ with 64 scans.

*Monosaccharide composition analysis*: LP (5.0 mg) was hydrolyzed in 2 mL of 4 M trifluoroacetic acid at 100 °C for 6 h and then dried under a nitrogen stream. The resulting residue was co-evaporated to dryness with 2 mL of methanol, and this step was repeated four times. The dried hydrolysate was reconstituted in 2 mL of distilled water and then subjected to PMP derivatization. The derivatized solution was filtered through a 0.22 μm membrane prior to analysis. Chromatographic separation was carried out on a C18 column (150 mm × 3 mm) using a high-performance liquid chromatography system (LC-20T, Agilent, Santa Clara, CA, USA). Mobile phase A consisted of 15% acetonitrile in 0.05 M phosphate buffer (pH 6.9), and mobile phase B consisted of 40% acetonitrile in 0.05 M phosphate buffer (pH 6.9). The injection volume was set at 20 μL. The flow rate was maintained at 0.8 mL/min, and the column temperature was regulated at 25 °C.

*NMR analysis*: LP (50 mg) was dissolved in 0.5 mL D_2_O and then transferred to an NMR tube. One-dimensional NMR spectra (^1^H and ^13^C) and two-dimensional NMR (1H-1H COSY, HSQC, and HMBC) spectra were recorded on an NMR spectrometer (Ascend^TM^ 400, Bruker, Billerica, MA, USA).

### 4.5. Gene Expression Analysis

Bone marrow-derived dendritic cells (BMDCs) were seeded on a 6-well plate (10^6^ cells per well) and then incubated with lipopolysaccharide (LPS, 100 ng/mL) or LP (100 μg/mL). After 24 h incubation, the cells were washed. Total RNA was isolated from the BMDCs using a commercially available RNA extraction kit (Vazyme, Nanjing, China). The expression levels of *Tnf*, *Il12a* and *Il1b* were detected by using qPCR (QuantStudio 7 Flex, Thermo Fisher Scientific, USA).

### 4.6. Cytokine Analysis

BMDCs were seeded on a 6-well plate (10^6^ cells per well) and subsequently incubated with LPS (100 ng/mL) or LP (100 μg/mL). After 24 h incubation, the supernatant was collected, and the levels of pro-inflammatory cytokines (TNF-α, IL12p70 and IL1β) were determined using ELISA kits (Invivogen, San Diego, CA, USA).

### 4.7. Detection of CD80 and CD86

BMDCs were seeded on a 6-well plate at a density of 10^6^ cells per well and then treated with LP at a concentration of 100 μg/mL for 24 h. After incubation, the cells were washed, collected via centrifugation (300× *g*), and resuspended in phosphate-buffered saline (PBS) containing 0.5% bovine serum albumin (BSA). Subsequently, they were stained with PE-conjugated anti-CD80, PE-conjugated anti-CD86, and PE-Cy7-conjugated anti-CD11c for 30 min and analyzed by flow cytometry (Beckman Coulter CytoFLEX S, Brea, CA, USA).

### 4.8. Cellular Uptake of OVA

To investigate the effect of LP on the cellular uptake of OVA, FITC-labeled OVA was added to the BMDCs with or without LP. The proportion of FITC-OVA^+^ BMDCs was detected by flow cytometry (Beckman Coulter CytoFLEX S, Brea, CA, USA). The median fluorescence intensity of FITC-OVA^+^ BMDCs was calculated.

### 4.9. Animal Immunization

Fifteen 8-week-old female C57BL/6J mice were randomly divided into three groups (OVA, Alum+OVA, and LP+OVA) by random number generator. Each group contained 5 mice. All immunizations were administered subcutaneously via hind footpad injection using an insulin syringe. In the OVA group, each mouse received 10 μg of OVA. In the Alum+OVA group, each mouse received 10 μg of OVA and 200 μg of aluminum hydroxide, while in the LP+OVA group, each mouse received 10 μg of OVA along with 200 μg of LP. The primary immunization was conducted on Day-0, and a booster immunization was conducted on Day-14. Animals that suffered 20% weight loss or persistent pain after immunization were excluded from the experiment.

### 4.10. Detection of Humoral and Cellular Immune Responses

To detect the humoral response, blood samples were obtained from the angular vein on Day-14 and Day-28, and serum was collected by centrifugation for antibody detection. The levels of IgG, IgG1 and IgG2c were detected using an ELISA kit (Chondrex, Woodinville, WA, USA).

To detect the cellular immune response induced by LP+OVA, splenocytes were harvested on Day-56 and stimulated with an OVA-specific, MHC-I-restricted peptide (OVA 257-264: SIINFEKL) or an OVA-specific, MHC-II-restricted peptide (OVA 323-339: ISQAVHAAHAEINEAGR) for 24 h. The proportion of CD4^+^ T cells and CD8^+^ T cells with IFN-γ production were determined by flow cytometry (Beckman Coulter CytoFLEX S, Brea, CA, USA).

### 4.11. Histopathological Analysis

At the experimental endpoint (Day-56), the mice were euthanized by CO_2_, and the heart, liver, spleen, lungs, and kidneys were immediately harvested for histopathological analysis. The tissues were fixed in 4% paraformaldehyde for 48 h at room temperature and then processed routinely. They were dehydrated through a graded ethanol series, cleared in xylene, and embedded in paraffin wax. Serial sections with a thickness of 6 µm were cut using a rotary microtome and mounted on glass slides. For staining, the sections were deparaffinized, rehydrated, and subjected to a standard hematoxylin and eosin (H&E) staining protocol. Briefly, the nuclei were stained with Mayer’s hematoxylin for 1 min, rinsed in tap water to develop the blue color, and differentiated in 1% acid alcohol. Cytoplasmic counterstaining was performed with eosin Y for 1 min. Finally, the sections were dehydrated through an ascending ethanol series, cleared in xylene, and permanently mounted using a synthetic resin medium for microscopic examination (Leica DMI8, Wetzlar, Germany).

### 4.12. Statistical Analysis

Results are presented as mean ± standard deviation (SD). Statistical analysis was performed using Prism software (GraphPad), wherein the normality and homogeneity of variance were assessed. A sample size of n = 3 was used for in vitro experiments and a sample size of n = 5 for in vivo experiments. Differences between groups were evaluated by means of one-way ANOVA with Tukey’s post hoc test.

## 5. Conclusions

In this work, a novel LP was extracted from the pulp of *Dimocarpus longan* Lour. through the MIEF-TPP method. The monosaccharide composition, combined with NMR spectra analysis, revealed that the primary structure of LP was (→6)-α-Glcp-(1→), with a side chain consisting of glucose residue linked by an α-1,4-glycosidic bond. In vitro experiments indicated that LP could induce the secretion of pro-inflammatory cytokines and expression of costimulatory molecules in BMDCs through activating TLR4. More importantly, LP could promote antigen cross-presentation when formulated with OVA. In vivo experiments demonstrated that LP+OVA significantly enhanced both humoral and cellular immune responses without causing adverse effects to main organs. Therefore, our findings indicate that LP can serve as a highly effective and safe adjuvant, underscoring its potential for vaccine development.

## Data Availability

All data are contained within the article.

## Supplementary Materials

The following supporting information can be downloaded at: https://www.mdpi.com/article/10.3390/ijms27093980/s1.
