# Supplementary material for: Polysaccharide Extracted from Longan (Dimocarpus longan Lour.) as Novel Adjuvant to Boost Humoral and Cellular Immune Responses
Source: Int J Mol Sci. 2026 Apr 29;27(9):3980. doi: 10.3390/ijms27093980 (PMC13163353; doi:10.3390/ijms27093980)
Supplement: Supplementary file 1 [file ijms-27-03980-s001.zip › ijms-4255219-supplementary.pdf]

Supplementary Material

Table S1. Chemical shift in ^{1}H and ^{13}C in residue A and residue B of LP

	H-1/C-1	H-2/C-2	H-3/C-3	H-4/C-4	H-5/C-5	H-6/C-6
Residue A	4.95/97.81	3.56/71.37	3.69/73.30	3.49/69.43	3.89/70.13	3.96/65.39
Residue B	5.22/98.00	3.40/69.44	3.60/73.24	3.45/63.71	3.87/69.18	3.92/65.45

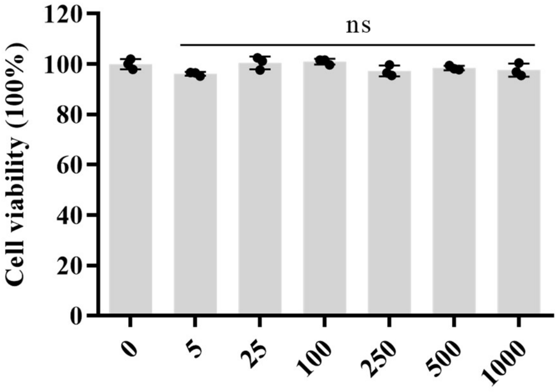

Figure S1. Viability of BMDC treated with LP at different concentrations for 24 h. n = 3, values represent means ± standard deviation. Statistical significance was calculated by one-way ANOVA with a Tukey's post hoc test. ns: no significance; ∗p < 0.05, ∗∗p < 0.01, ∗∗∗p < 0.001, ∗∗∗∗p < 0.0001.

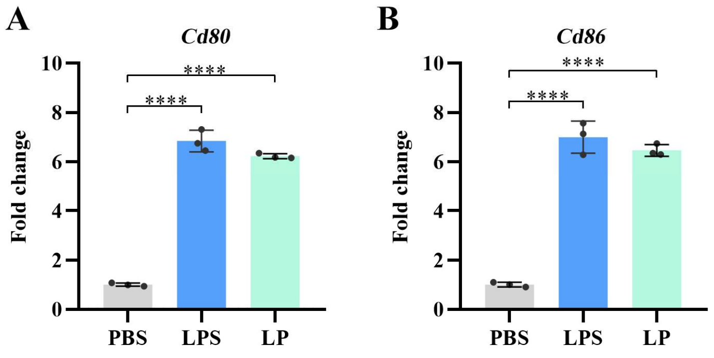

Figure S2. Gene expression of *Cd80* (A), *Cd86* (B) BMDCs treated with LP at a concentration of 100 μg/mL. LPS was used as a control, with a concentration of 100 ng/mL. n = 3, values represent means ± standard deviation. Statistical significance was calculated by one-way ANOVA with a Tukey's post hoc test. ns: no significance; ∗p < 0.05, ∗∗p < 0.01, ∗∗∗p < 0.001, ∗∗∗∗p < 0.0001.
